# Small doses of epinephrine prolong the recovery from a rocuronium-induced neuromuscular block: a case report

**DOI:** 10.1186/s12871-018-0544-2

**Published:** 2018-07-11

**Authors:** Hubert J. Schmitt

**Affiliations:** Department of Anaesthesia, Friedrich-Alexander University Erlangen-Nuremberg, Krankenhausstrasse 12, D-91054 Erlangen, Germany

**Keywords:** Catecholamines, Epinephrine, Neuromuscular blocking agents, Rocuronium

## Abstract

**Background:**

During anaesthesia it is not uncommon to administer epinephrine in patients blocked by non-depolarizing muscle relaxants. However, there are few reports on possible interaction of epinephrine with neuromuscular transmission in humans.

**Case presentation:**

An otherwise healthy 74-yr-old man underwent transurethral resection of a benign prostatic hyperplasia under total intravenous anaesthesia. Because of repeated drop in heart rate and blood pressure the patient received in total three bolus of epinephrine 5 μg, respectively. Each time this small dose of epinephrine intensified a rocuronium-induced neuromuscular block verified by acceleromygraphy. Further anaesthetic course was uneventful.

**Conclusions:**

In this case reported here small doses of intravenously administered epinephrine markedly prolonged a rocuronium-induced neuromuscular block. Given the widely used co-administration of epinephrine and muscle relaxants possible adrenergic interference with neuromuscular transmission would have implications for daily anaesthetic practice.

## Background

Arterial hypotension and/or bradycardia often occur during anaesthesia despite adequate fluid administration and careful anaesthetic drug titration. This situation calls for the administration of sympathomimetic amines to maintain sufficient arterial pressure. Although the effects of different catecholamines on haemodynamic variables are well documented [1] few reports are available on potential adrenergic effects at the neuromuscular junction in humans. An intensification of a rocuronium-induced neuromuscular block (NMB) by epinephrine was reported twice, once in association with co-administration of lidocaine [2] and once by the use of nebulized epinephrine [3].

In this clinical case report a patient is presented where the administration of single small doses of epinephrine altered the recovery characteristic of a rocuronium-induced NMB.

## Case presentation

This case report was prepared after obtaining the patient’s written informed consent. The local Ethics Committee of the Friedrich-Alexander University of Erlangen approved the publication of this report (protocol number Nr. 171_15 Bc). The data was recorded within the context of an observational study investigating the recovery characteristics of a rocuronium-induced NMB during urological surgery (data not published).

A 74-yr-old male (height, 174 cm; weight, 81 kg, ASA physical status 1) was scheduled for transurethral resection of a benign prostatic hyperplasia under general anaesthesia. The patient had never undergone anaesthesia before. He had no relevant medical history and was in good health. His only medication was the alpha-blocker tamsulosin for pharmacological treatment of benign prostatic hypertrophy. Physical examination did not show any anomalies, preoperative electrocardiogram showed sinus rhythm, and a pulmonary function test revealed normal values.

**Fig. 1:**
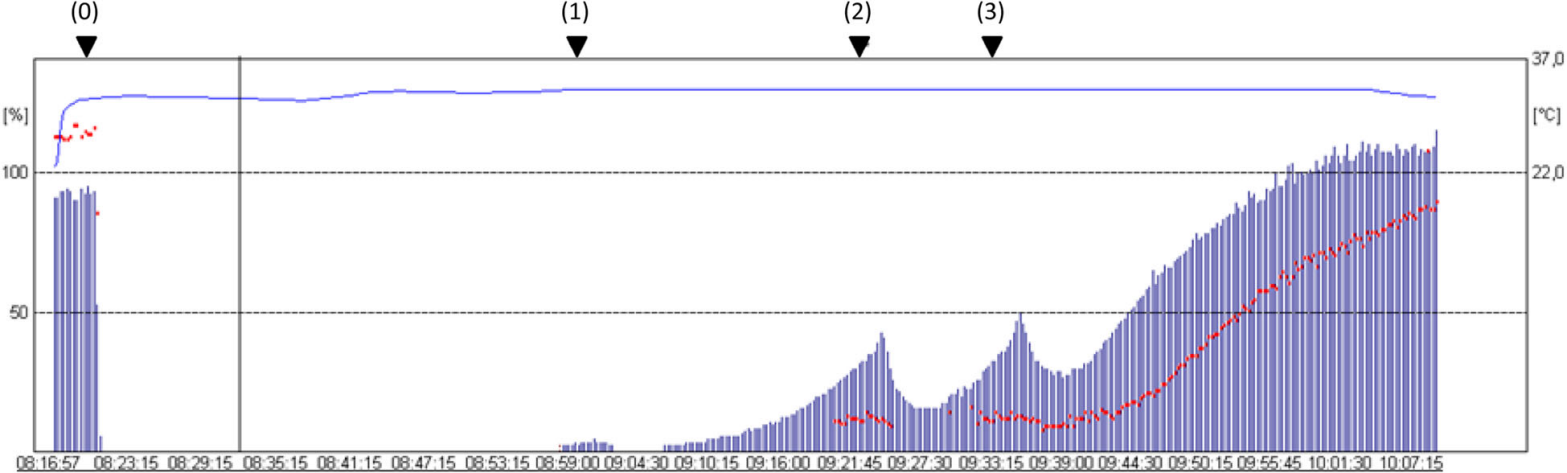
Original record shows complete recovery characteristic after a single bolus of rocuronium. At time 0 (arrow numbered 0) rocuronium 0.6 mg/kg was given. The arrows 1 to 3 indicate time of administration of a single bolus of epinephrine 5 μg, respectively. Columns show the T1 value (%), the dotted line the TOF ratio (%). The continuous line represents actual skin temperature (degree Celsius)

After written informed consent the patient was premedicated with orally dikaliumchlorazepat 20 mg. On arrival at the theatre the patient showed a heart rate (HR) of 63 beats min^− 1^ (bpm) and blood pressure (BP) (systolic/diastolic//mean) of 115/70//85 mmHg, venous blood sample revealed normal sodium and potassium values. Following pre-oxygenation, anaesthesia was induced with propofol (1.5 mg kg^− 1^) and fentanyl (0.2 μg kg^− 1^). After establishing adequate mask ventilation the right arm was prepared for routine neuromuscular monitoring using acceleromyography. The ulnar nerve was stimulated supramaximal with repeated train-of-four (TOF) stimuli (2HZ, 0.2 ms duration at 15 s intervals) deliberated by surface electrodes placed above the wrist. The transducer was fixed tightly at the distal interphalangeal joint of the thumb. The monitoring arm was kept free from IV infusion and an arterial pressure cuff. The TOF monitor was connected to a portable PC with online data recording and processing (TOF-Watch© SX Monitor program, Organon, Germany). After calibration and initial signal stabilization of control response a single dose of rocuronium 50 mg was administered IV over 5 s, and the trachea intubated awaiting complete NMB. At that point BP was 121/74//89 mmHg and HR at 75 bpm. Anaesthesia was maintained with IV infusion of propofol, the lungs ventilated with oxygen/nitrous oxide. Following induction of anaesthesia the mean arterial pressure dropped slightly but increased above pre-induction level with the beginning of surgery. At this point the remifentanil infusion was started at a rate of 0.35 μg/kg/min. Despite careful titration of remifentanil (0.15 μg/kg/min) HR and BP dropped continuously over the next minutes. Remifentanil infusion was briefly stopped and atropine 0.8 mg was administered but without any effect. As BP dropped to 75/45//55 mmHg and HR to 39 bpm a single bolus of epinephrine 5 μg was administered intravenously. Over the next minutes HR increased to 62 bpm and BP to 115/67//83 mmHg. The moment epinephrine was given, the first twitch response (T_1_) had recovered to 3%. Three minutes after epinephrine administration the T_1_ response disappeared completely and returned not until another 4 min (Fig. 1 - arrow 1). During the course of anaesthesia administration of additional two boluses of epinephrine (5 μg) were necessary to maintain HR above 35 bpm and mean arterial pressure above 60 mmHg (Fig. 1-arrow 2: BP 75/43//53 mmHg HR 37 bpm; arrow 3: BP 73/42//51, HR 36 bpm). Initially, in both instances the administration of epinephrine induced an increase of the first and the second twitches within two minutes for a short time (Fig. 1). At both time points however, this increase was followed by a decrease of T_1_ from 33 to 16% and from 33 to 27%, respectively. The T_1_ value returned to its pre-epinephrine level after 10 and 7 min, respectively. Consistently the TOF–ratio decreased at the second and third epinephrine administration (Fig. 1 – arrow 2 and 3). The recovery times are summarized in Table 1. The patient’s skin temperature remained constant throughout the complete monitoring course between 32 and 33 °C. The TOF ratio recovered to 90% not until 111 min after rocuronium administration. Further anaesthetic course was uneventful. After gaining spontaneous breathing the tracheal tube was removed and the patient transferred to the recovery room.

**Table 1:** Recovery times after three doses of epinephrine

Recovery characteristics	Time (min)
Rocuronium administraton	0
T1 25%	71
T1 50%	84
T1 75%	89
T1 90%	94
T1 100%	98
TOF 90%	111

## Discussion and conclusions

This case report demonstrates a clinically marked alteration of the recovery characteristic of a rocuronium-induced NMB by small doses of intravenously administered epinephrine. Single boluses of epinephrine caused a remarkable prolonged duration of a rocuronium-induced NMB compared to published data [4].

The marked hemodynamic changes in this patient during the course of anaesthesia cannot be explained satisfactorily. Tamsulosin like other alpha_1_-adrenergic-receptorantagonists may cause hypotension by vasodilatation [5]. This could explain the recorded hypotension. The bradycardia might have been caused by remifentanil a known side-effect of this drug.

The administration of a single low dose of epinephrine (2 to 8 μg) to modestly increase HR and systolic pressure is a well-established technique [1]. At low concentrations epinephrine act primarily stimulating beta adrenergic receptors at the heart and vasculature. In contrast to these well documented effects there are hardly any reports regarding possible interaction of catecholamines with neuromuscular transmission in humans. This fact is somewhat surprising since early experimental studies revealed different effects of sympathomimetic amines on neuromuscular transmission. In an experiment with curarized striated muscle a significant prolongation of a NMB was shown by a small dose of adrenaline [6]. Successive experiments confirmed this enhancement of a NMB by several catecholamines. These reports documented a short augmentation of twitch response (“anti-curare effect”) by adrenaline followed by a varying duration of block potentiation depending on adrenaline dosage [7]. This short “anti-curare effect” can be explained by an enhanced transmitter release from the motor nerve. Experimental studies demonstrate that this transmitter release can be mediated via presynaptic alpha- and beta adrenoceptors [8–10], These experiments also show that site of action at the neuromuscular junction vary between different catecholamines. Sympathomimetic amines increase transmitter output from the motor nerve markedly and change the resistance of the post-synaptic membrane [11].

In the presented case epinephrine was administered during the recovery period from a rocuronium-induced NMB. Small doses of epinephrine induced a short augmentation of twitch response each time followed by twitch inhibition of several minutes. These recorded dual actions of adrenaline in the reported case strongly resemble the result of several experimental studies [7].

One can only speculate as to the pathophysiological mechanism behind the recorded transient enhancement of the rocuronium-induced NMB by epinephrine. Several theories are imaginable: Changes in tissue blood supply, pharmacokinetic drug interactions with changes in distribution, protein binding, clearance, or metabolism of rocuronium. However, the observed immediate effect by epinephrine administration cannot be explained by short-term modifications of rocuronium pharmacokinetics or pharmacodynamics. It was such a small dose of epinephrine that it did not even change skin temperature indicating that the used dose did act only on beta adrenergic receptors (Fig. 1). Another explanation could be a possible influence of epinephrine on the acetylcholine esterase activity but this is purely speculative.

As well, possible interaction with transmitter at the neuromuscular junction should also to be considered. Like other catecholamines epinephrine exerts its pharmacologic effect by the generation of cyclic AMP, a well described second messenger. Experimental evidence points to ATP and its derivatives modulating neuromuscular transmission [9, 12, 13]. Whether these mechanisms might have played a role in the observation reported in this case cannot be clarified.

Our case describes another example of possible enhancement of rocuronium-induced NMB by epinephrine. It is most likely that the reported decrease of TOF ratio by Ninomiya et al. was due to epinephrine [2], because a following investigation showed no impact of lidocaine on rocuronium-induced block [14]. Another report by Arndt et al. of postoperative reparalysis following nebulized epinephrine also highlights the assumed interaction [3]. Considering our data, previous clinical reports, and experimental findings all this indicates that epinephrine caused the documented prolongation of the recovery period in our patient.

In conclusion this report documents possible modulation of the recovery characteristics of a rocuronium-induced NMB by low dose of epinephrine. Given the widely used co-administration of catecholamines and muscle relaxants during anaesthesia possible adrenergic interactions during neuromuscular transmission would have implications for daily anaesthetic practice. Further investigations are necessary to elucidate causal mechanisms.

## Abbreviations

AMP: adenosine monophosphate
ATP: adenosine triphosphate
BP: blood pressure
BPM: beats min^− 1^
HR: heart rate
NMB: neuromuscular block
TOF: train-of-four
